# The Relationship Between Serum SFRP5, ApoA‐I, HDL3‐C Level and In‐Stent Restenosis After PCI in Acute Myocardial Infarction and the Combined Predictive Value

**DOI:** 10.1002/kjm2.70000

**Published:** 2025-03-08

**Authors:** Li‐Qiang Cui, Xue‐Dong Wang

**Affiliations:** ^1^ Department of Cardiology II General Hospital of Fuxin Mining Industry Group of Liaoning Health Industry Group Fuxin Liaoning P. R. China

**Keywords:** acute myocardial infarction, apolipoprotein A‐I, in‐stent restenosis, percutaneous coronary intervention, secreted frizzled‐related protein 5

## Abstract

This study aims to investigate the relationship between serum secreted frizzled‐related protein 5 (SFRP5), apolipoprotein A‐I (ApoA‐I), high‐density lipoprotein 3‐cholesterol (HDL3‐C) and in‐stent restenosis (ISR) after percutaneous coronary intervention (PCI) in acute myocardial infarction (AMI) and their combined predictive value. The clinical data of 128 AMI patients who underwent PCI in our hospital from July 2020 to July 2023 were retrospectively analyzed. After 12 months of follow‐up, the patients were divided into the ISR group (24 cases) and the non‐ISR group (104 cases) according to the results of coronary angiography. The 24 patients with ISR were divided into Grade III (lumen stenosis area of 50%–70%, 15 cases) and Grade IV (lumen stenosis area of 76%–100%, 9 cases). The general data of the two groups were compared. The serum levels of SFRP5, ApoA‐I, and HDL3‐C in the two groups were analyzed on the second day after the operation. The levels of SFRP5, ApoA‐I, and HDL3‐C in patients with different degrees of stenosis were compared. The correlation between serum SFRP5, ApoA‐I, HDL3‐C levels and ISR after PCI was analyzed by bivariate correlation Kendall tau‐*b* (K). Logistic regression was used to analyze the influencing factors of ISR after PCI. The receiver operating characteristic (ROC) curve was drawn to analyze the predictive value of SFRP5, ApoA‐I, and HDL3‐C in ISR after PCI. The proportion of patients with diabetes and a smoking history in the ISR group was higher than that in the non‐ISR group. The stent length (29.52 ± 5.47 mm) and hs‐CRP level (3.38 ± 0.51 mg/L) in the ISR group were higher than those in the non‐ISR group (23.56 ± 5.37 mm and 2.78 ± 0.52 mg/L) (*p* < 0.05). SFRP5 (15.33 ± 2.60 ng/mL), ApoA‐I (1.22 ± 0.37 g/L) and HDL3‐C (0.31 ± 0.07 mmol/L) in the ISR group were higher than those in the non‐ISR group (19.79 ± 3.09 ng/mL, 1.77 ± 0.41 g/L, and 0.46 ± 0.11 mmol/L) (*p* < 0.001). The levels of SFRP5 (17.57 ± 2.57 ng/mL), ApoA‐I (1.56 ± 0.34 g/L) and HDL3‐C (0.36 ± 0.07 mmol/L) in the Grade III group were higher than those in the Grade IV group (13.15 ± 2.35 ng/mL, 0.98 ± 0.20 g/L, and 0.25 ± 0.05 mmol/L) (*p* < 0.05). The results of bivariate correlation Kendall tau‐*b* (K) analysis showed that the levels of serum SFRP5, ApoA‐I, and HDL3‐C were negatively correlated with ISR (*r* < 0, *p* < 0.05). Logistic regression analysis showed that diabetes and hs‐CRP were risk factors for ISR after PCI (OR > 1, *p* < 0.05). SFRP5, ApoA‐I, and HDL3‐C were protective factors for ISR after PCI (OR < 1, *p* < 0.05). The ROC curve showed that the AUC of SFRP5, ApoA‐I, and HDL3‐C levels alone and in combination to predict ISR in AMI patients after PCI was > 0.70, which had certain predictive value, and the combined value was higher. In conclusion, diabetes and high levels of hs‐CRP were risk factors for ISR in patients with AMI after PCI. High levels of SFRP5, ApoA‐I, and HDL3‐C were protective factors for ISR after PCI, and their combined detection had certain value in predicting ISR after PCI. This would provide guidance strategy for clinical timely intervention measures to reduce the occurrence of ISR in AMI patients after PCI.

## 1 Introduction

The incidence and mortality of acute myocardial infarction (AMI) are high worldwide, and the incidence rate is increasing year by year [1]. The continuous development of AMI will lead to the decline of cardiac function and cardiac and systemic blood circulation disorders, and some patients can directly experience shock, which brings a great threat to the life safety of patients [2]. The clinical treatment of AMI is mainly based on surgery and drug therapy. Percutaneous coronary intervention (PCI) is one of the effective treatment methods, which can effectively unblock the occluded coronary arteries, improve myocardial function, and reduce mortality [3]. However, there is still a chance of in‐stent restenosis (ISR) after PCI surgery. Therefore, it is very important to analyze the influencing factors of ISR in AMI patients after PCI to prevent ISR.

Serum Secreted Curl Associated Protein 5 (SFRP5) is an anti‐inflammatory adipokine, which can reduce chronic inflammatory conditions by inhibiting the Wnt signaling pathway and has a regulatory effect on atherosclerosis and cardiovascular diseases. Previous studies have shown that SFRP5 can inhibit the occurrence and development of cardiovascular diseases [4]. High‐density lipoprotein (HDL) can inhibit atherosclerosis by promoting the reverse transport of cholesterol and reducing the deposition of cholesterol on the vascular wall, which may have a positive significance in improving cardiovascular stenosis [5]. Apolipoprotein A‐I (ApoA‐I) is the main protein in HDL, and it is also the main material basis of high‐density lipoprotein cholesterol (HDL‐C) in anti‐atherosclerosis, which is closely related to the occurrence and development of atherosclerosis. High‐density lipoprotein 3‐cholesterol (HDL3‐C) is one of the important subtypes of HDL‐C, which has anti‐atherosclerotic effects such as cholesterol reverse transport, anti‐inflammatory, and anti‐oxidant, and is closely related to the progress of cardiovascular disease [6]. The occurrence of ISR may be related to intimal neogenesis, atherosclerosis, inflammatory response, and other factors. It can be seen that SFRP5, ApoA‐I, and HDL3‐C might be related to the occurrence and development of ISR.

This study focuses on analyzing the relationship between serum SFRP5, ApoA‐I, HDL3‐C, and ISR in AMI patients after PCI and their combined predictive value.

## 2 Materials and Methods

### 2.1 The Inclusion of Study Subjects

**FIGURE 1:**
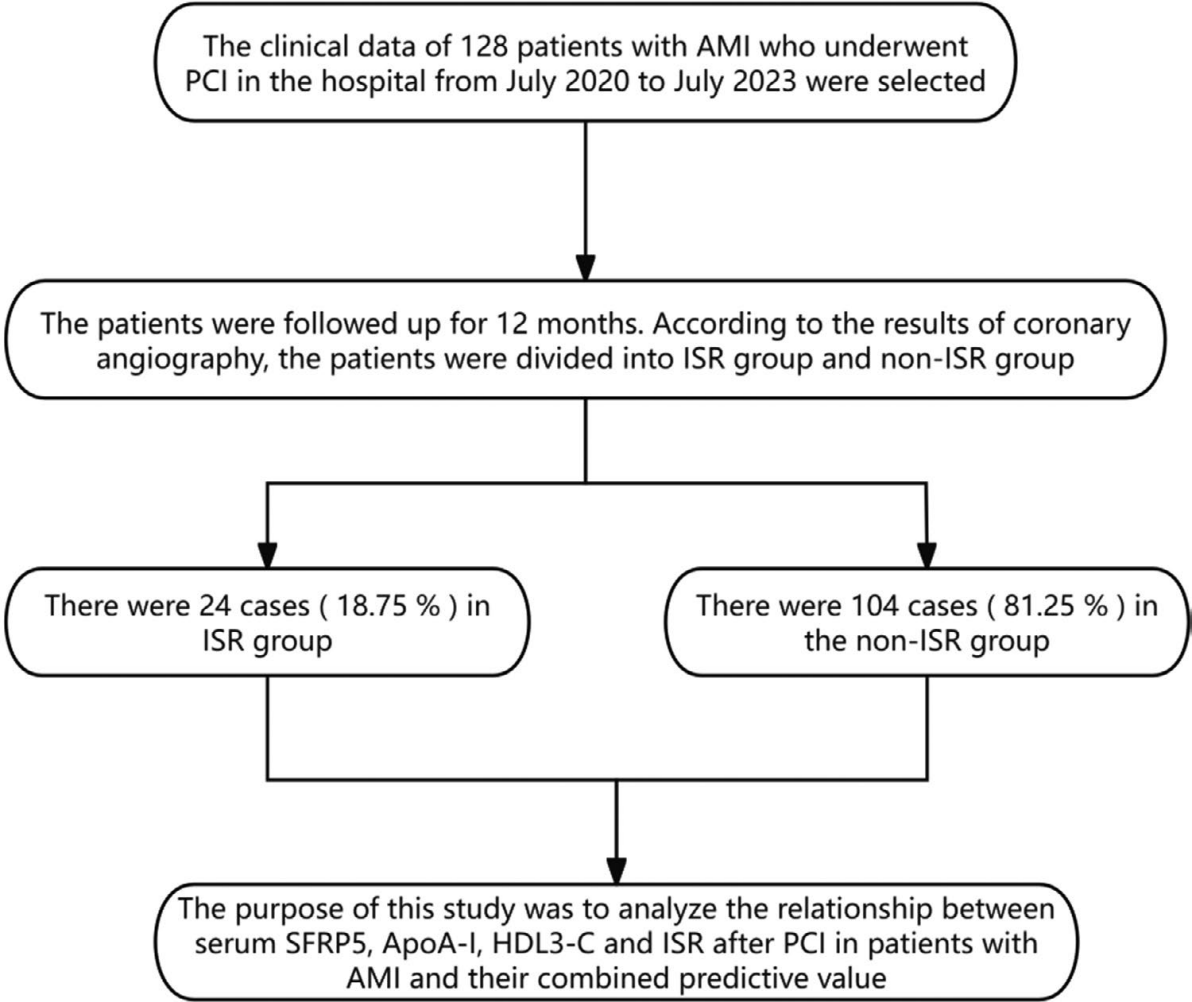
The inclusion process of general information. The consecutive patients were analyzed during 3 years. Patients were selected by inclusion and exclusion criteria.

The clinical data of 128 AMI patients who underwent PCI in our hospital from July 2020 to July 2023 were retrospectively analyzed. Inclusion criteria: (1) Patients who met the diagnostic criteria for AMI [7] and were examined by electrocardiogram and cardiac biomarkers. (2) The age of patients ≥ 18 years. (3) Patients successfully underwent PCI surgery. (4) Patients underwent coronary angiography at 12 months after PCI. (5) The clinical data of the patients were complete. Exclusion criteria: (1) Patients with severe liver and renal insufficiency. (2) Patients with malignant tumors. (3) Patients with a previous history of PCI and coronary‐artery bypass grafting. (4) Patients with rheumatic heart disease, valvular heart disease, cardiogenic shock, and other heart diseases. (5) Patients with autoimmune diseases. (6) Patients with severe infectious diseases and coagulopathy. (7) Patients with mental or conscious disorders. This study was approved by The Ethics Committee of Fuxin Mining General Hospital (2023011). Written informed consent was obtained from participants for participation in the study and all methods were carried out in accordance with relevant guidelines and regulations. This study complied with the principles of Medical Ethics and was ratified by the Ethics Committee of our hospital. The general data selection process was shown in Figure 1.

### 2.2 Methods

#### 2.2.1 General Information

The data of age, gender, body mass index (BMI), diabetes, hypertension, stroke history, smoking history, drinking history, number of lesion vessels, infarct‐related vessels, number of implanted stents, stent diameter, and stent length were collected before discharge.

#### 2.2.2 The Detection of Inflammatory Factor Levels

On the premise of obtaining the consent of patients for blood sample collection, 2 mL of fasting elbow vein blood was collected on the second day after PCI, centrifuged at 3500 r/min for 10 min with a radius of 10 cm, and the upper serum was collected. The levels of high‐sensitivity C‐reactive protein (hs‐CRP), interleukin‐6 (IL‐6) and tumor necrosis factor alpha (TNF‐α) were detected by enzyme‐linked immunosorbent assay (ELISA). The level of low‐density lipoprotein cholesterol (LDL‐C) was detected by automatic biochemical analyzer KS‐380 (Shandong Kelissen Biological, Lu mechanical injection standard 20,212,220,956).

#### 2.2.3 Detection of Cardiac Function Indicators

Left ventricular ejection fraction (LVEF), left ventricular end‐diastolic diameter (LVDD) and left ventricular end‐systolic diameter (LVSD) were measured by color Doppler ultrasound diagnostic instrument Apogee 3800 (Shantou Ultrasonic Instrument Research Institute, National Mechanical injection Standard 20,233,060,164) on the second day after PCI.

#### 2.2.4 Detection of Serum SFRP5, ApoA‐I, and HDL3‐C Levels

On the second day after PCI, all patients were fasted for more than 12 h, and 3 mL of fasting elbow vein blood was collected in the morning. The upper layer of serum was centrifuged and stored in the refrigerator at −80°C.

The serum level of SFRP5 was detected by ELISA. Briefly, antigens were coated on microplate plates and incubated in an incubator at37°C for 1–2 h before being washed to remove unbound antigens. Blocking solution was added and, after 30 min of incubation, unbound blocking solution was removed by washing. Standards or samples to be tested were added, and after 1 h of incubation, unbound proteins were removed by washing. Enzyme‐labeled antibody was added, and after 1 h of incubation, unbound enzyme‐labeled antibody was removed by washing. An appropriate amount of substrate solution was added and incubated for 15–30 min. After observing color changes, termination solution was added. The optical density values of each well were measured on a microplate reader, and the data were recorded and analyzed.

The level of ApoA‐I was detected by immunoturbidimetry. Antigen–antibody reactions were performed using anti‐ApoA‐I antibodies and ApoA‐I in the samples. After the completion of the reaction, changes in absorbance were measured by transmission turbidity to reflect the concentration of ApoA1.

HDL3‐c level was detected by the polyethylene glycol 20,000 precipitation method. 170 g of polyethylene glycol 20,000 was dissolved in 0.1 mol/L phosphate buffer (pH 7.5) and added to 1 L as a precipitant. Hundred microliters of serum was mixed with 200 μL precipitant, left at room temperature for 10 min, centrifuged at 3000 r/min for 20 min, and the serum was purified. Hundred microliters of the supernatant was mixed with 1.0 μL of the enzyme reagent to detect HDL3‐c levels.

### 2.3 Determination Criteria and Grouping Methods for ISR

ISR judgment criteria [8]: Quantitative analysis of coronary angiography showed that the target vessel stenosis within 5 mm of the stent site or its edge was ≥ 50%. ISR grading standard [8]: Grade I: Lumen stenosis area < 25%; Grade II: Lumen stenosis area was 26%–50%; Grade III: Lumen stenosis area was 50%–70%; Grade IV: Lumen stenosis area was 76%–100%. Grades III and IV were considered as ISR. All patients underwent PCI by the same intervention team. All patients were followed up for 12 months and rechecked once every 3 months. Laboratory indicators such as blood routine, coagulation function, and electrocardiogram were examined. Further coronary angiography was performed if abnormalities were found. According to the results of coronary angiography, 128 patients were divided into ISR group (24 cases accounting for 18.75%) and non‐ISR group (104 cases accounting for 81.25%). The 24 patients with ISR were further divided into Grade III (15 cases accounting for 62.50%) and Grade IV (9 cases accounting for 37.50%).

### 2.4 Statistical Analysis

SPSS 25.0 statistical software was used for data analysis. Measurement data was expressed using with $\bar{x}\pm s$, and independent sample *t*‐test was used for inter group comparison. Enumeration data were expressed as *n* (%) and compared using *χ* ^2^ test. Bivariate correlation Kendall tau‐*b* (K) analysis was used to analyze the relationship between ISR and the changes of SFRP5, ApoA‐I, and HDL3‐C levels. Binary logistic regression was used to analyze the influencing factors of ISR in AMI patients after PCI. Receiver operating characteristic (ROC) curve was drawn and the area under the curve (AUC) was calculated to analyze the predictive value of SFRP5, ApoA‐I, and HDL3‐C levels for ISR in AMI patients after PCI. *p* < 0.05 was considered as statistically significant.

## 3 Results

### 3.1 General Information

After 12 months of follow‐up, the average time of ISR was 8.63 ± 1.14 months. The proportion of patients with diabetes and a smoking history in the ISR group was higher than that in the non‐ISR group. The stent length (29.52 ± 5.47 mm) and hs‐CRP (3.38 ± 0.51 mg/L) in the ISR group were higher than those in the non‐ISR group (23.56 ± 5.37 mm and 2.78 ± 0.52 mg/L) (*p* < 0.05). There was no significant statistical difference in other data (*p* > 0.05, Table 1).

**TABLE 1:** Comparison of general materials ($\bar{x}\pm s$)/*n* (%).

Indicators		ISR group (*n* = 24)	Non‐ISR group (*n* = 104)	*t*/*χ* ^2^ value	*p*
Age (year)		62.66 ± 2.47	63.02 ± 2.57	0.623	0.535
Gender	Male	14 (58.33)	63 (60.58)	0.041	0.840
	Female	10 (41.67)	41 (39.42)		
BMI (kg/m^2^)		23.68 ± 1.21	23.89 ± 0.25	0.746	0.457
Diabetes	Yes	14 (58.33)	14 (13.46)	22.974	< 0.001
	No	10 (41.67)	90 (86.54)		
Hypertension	Yes	11 (45.83)	42 (40.38)	0.239	0.625
	No	13 (54.17)	62 (59.62)		
History of stroke	Yes	3 (12.5)	12 (11.54)	0.077	0.782
	No	21 (87.5)	92 (88.46)		
Smoking history	Yes	11 (45.83)	12 (11.54)	15.560	< 0.001
	No	13 (54.17)	92 (88.46)		
Drinking history	Yes	9 (37.5)	38 (36.54)	0.008	0.930
	No	15 (62.5)	66 (63.46)		
Number of stenosed coronary vessel	Single	8 (33.33)	36 (34.62)	0.088	0.957
	Double	10 (41.67)	40 (38.46)		
	Multiple	6 (25.00)	28 (26.92)		
Infarct related artery IRA	Front descending branch	12 (50.00)	49 (47.12)	0.237	0.888
	Left circumflex branch	4 (16.67)	15 (14.42)		
	Right coronary artery	8 (33.33)	40 (38.46)		
Type of stent	Sirolimus‐eluting stents	13 (54.17)	50 (48.08)	0.289	0.591
	Paclitaxel‐eluting stents	11 (45.83)	54 (51.92)		
Number of stents inserted (number)		1.86 ± 0.41	1.77 ± 0.40	0.989	0.325
Bracket diameter (mm)		3.03 ± 0.42	3.14 ± 0.44	1.113	0.268
Bracket length (mm)		28.50 ± 5.44	23.56 ± 5.37	4.018	< 0.001
hs‐CRP (mg/L)		3.38 ± 0.51	2.79 ± 0.51	5.019	< 0.001
IL‐6 (pg/mL)		10.52 ± 1.57	10.14 ± 1.43	1.152	0.252
TNF‐α (ng/mL)		3.52 ± 0.51	3.43 ± 0.53	0.755	0.452
LVEF (%)		59.04 ± 6.58	59.86 ± 6.26	0.573	0.568
LVDD (mm)		49.68 ± 2.31	48.62 ± 2.57	1.854	0.066
LVSD (mm)		37.21 ± 2.10	36.37 ± 2.17	1.709	0.088
LDL‐C (mmol/L)		2.35 ± 0.68	2.22 ± 0.64	0.887	0.377
Postoperative medication	Aspirin	23 (95.83)	101 (97.12)	0.106	0.745
	Statins	22 (91.67)	100 (96.15)	0.161	0.688
	β‐blocker	20 (83.33)	91 (87.50)	0.044	0.835
	ACEI	18 (75.00)	80 (76.92)	0.040	0.841
	Anticoagulation	5 (20.83)	18 (17.31)	0.012	0.912
	Diuretic	1 (4.17)	5 (4.81)	0.161	0.688

### 3.2 Comparison of Serum SFRP5, ApoA‐I, and HDL3‐C Levels Between Two Groups After Surgery

The level of SFRP5 (15.33 ± 2.60 ng/mL), ApoA‐I (1.22 ± 0.37 g/L) and HDL3‐C (0.31 ± 0.07 mmol/L) in the ISR group was lower than those in the non‐ISR group (19.79 ± 3.09 ng/mL, 1.77 ± 0.41 g/L, and 0.46 ± 0.11 mmol/L) (*p* < 0.001, Table 2).

**TABLE 2:** Comparison of serum SFRP5, ApoA‐I, and HDL3‐C levels between two groups after surgery ($\bar{x}\pm s$).

Groups	SFRP5 (ng/mL)	ApoA‐I (g/L)	HDL3‐C (mmol/L)
ISR group (*n* = 24)	15.33 ± 2.60	1.22 ± 0.37	0.31 ± 0.07
Non‐ISR group (*n* = 104)	19.79 ± 3.09	1.77 ± 0.41	0.46 ± 0.11
*t*	6.547	6.081	6.150
*p*	< 0.001	< 0.001	< 0.001

### 3.3 Comparison of SFRP5, ApoA‐I, and HDL3‐C Levels in Patients With Different Degrees of Stenosis

The levels of SFRP5 (17.57 ± 2.57 ng/mL), ApoA‐I (1.56 ± 0.34 g/L) and HDL3‐C (0.36 ± 0.07 mmol/L) in the Grade III group were higher than those in the Grade IV group (13.15 ± 2.35 ng/mL, 0.98 ± 0.20 g/L, and 0.25 ± 0.05 mmol/L) (*p* < 0.05, Table 3).

**TABLE 3:** Comparison of SFRP5, ApoA‐I, and HDL3‐C levels in patients with different degrees of stenosis ($\bar{x}\pm s$).

Groups	SFRP5 (ng/mL)	ApoA‐I (g/L)	HDL3‐C (mmol/L)
Grade III group (*n* = 15)	17.57 ± 2.57	1.56 ± 0.34	0.36 ± 0.07
Grade IV group (*n* = 9)	13.15 ± 2.35	0.98 ± 0.20	0.25 ± 0.05
*t*	4.208	4.517	4.025
*p*	< 0.001	< 0.001	< 0.001

### 3.4 The Correlation Between Serum SFRP5, ApoA‐I, HDL3‐C Levels With ISR After PCI in AMI Patients

**FIGURE 2:**
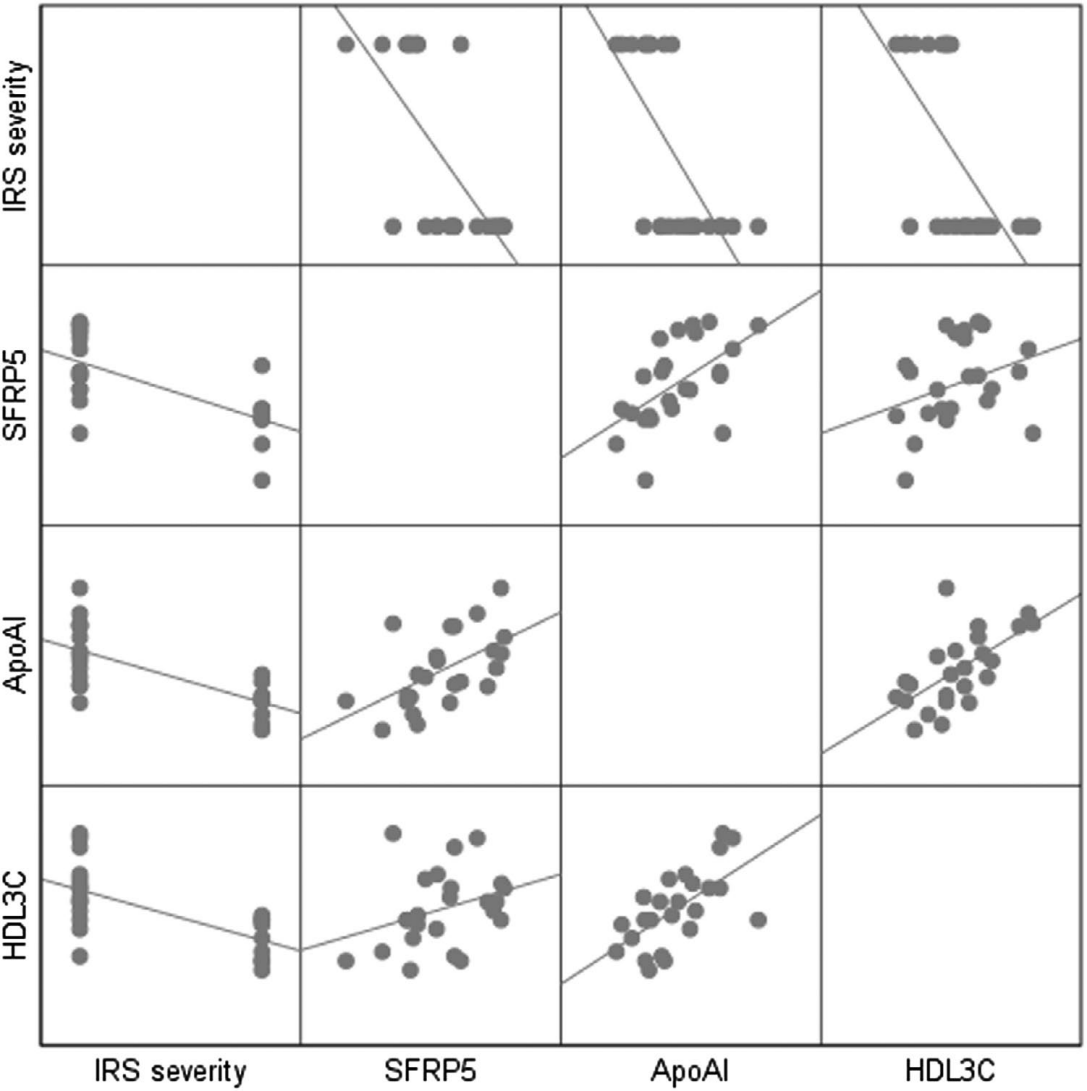
The correlation between serum SFRP5, ApoA‐I, and HDL3‐C levels with ISR after PCI in AMI patients.

The results of bivariate correlation Kendall tau‐*b* (K) analysis showed that the levels of serum SFRP5, ApoA‐I, and HDL3‐C were negatively correlated with ISR (*r* < 0, *p* < 0.05, Table 4 and Figure 2).

**TABLE 4:** The correlation between serum SFRP5, ApoA‐I, HDL3‐C levels with ISR after PCI in AMI patients *r* (*p*).

Indicators	SFRP5		ApoA‐I		HDL3‐C	
	*r*	*p*	*r*	*p*	*r*	*p*
ISR	−0.556	< 0.001	−0.586	< 0.001	−0.581	< 0.001

### 3.5 Analysis of the Influencing Factors of ISR in AMI Patients After PCI Surgery

The occurrence of ISR in AMI patients after PCI was taken as the dependent variable (“1” = ISR group, “0” = no ISR group), and the variables with statistically significant differences in Tables 1 and 2 were taken as covariates (diabetes and smoking history were classified variables, “1” = yes, “0” = no; stent length, hs‐CRP, SFRP5, ApoA‐I, HDL3‐C were continuous variables) for logistic regression analysis. The results showed that diabetes and hs‐CRP were risk factors, and SFRP5, ApoA‐I, and HDL3‐C were protective factors for ISR after PCI (OR < 1, *p* < 0.05, Table 5).

**TABLE 5:** Analysis of the influencing factors of ISR in AMI patients after PCI surgery.

Related factors	*β*	Standard error	*Waldχ* ^2^	*p*	OR	95% confidence interval
Smoking history	1.542	1.325	1.354	0.245	4.676	0.348–62.820
Diabetes	3.207	1.463	4.804	0.028	24.704	1.404–434.714
Bracket length	0.054	0.093	0.341	0.559	1.056	0.880–1.267
hs‐CRP	3.719	1.435	6.714	0.010	41.232	2.475–687.031
SFRP5	−0.588	0.299	3.870	0.049	0.555	0.309–0.998
ApoA‐I	−4.791	2.005	5.711	0.017	0.008	0.000–0.422
HDL3‐C	−19.317	8.932	4.677	0.031	< 0.001	0.000–0.164
Constant	8.321	6.867	1.468	0.226	—	—

### 3.6 The Predictive Value of SFRP5, ApoA‐I, and HDL3‐C for ISR After PCI in AMI Patients

**FIGURE 3:**
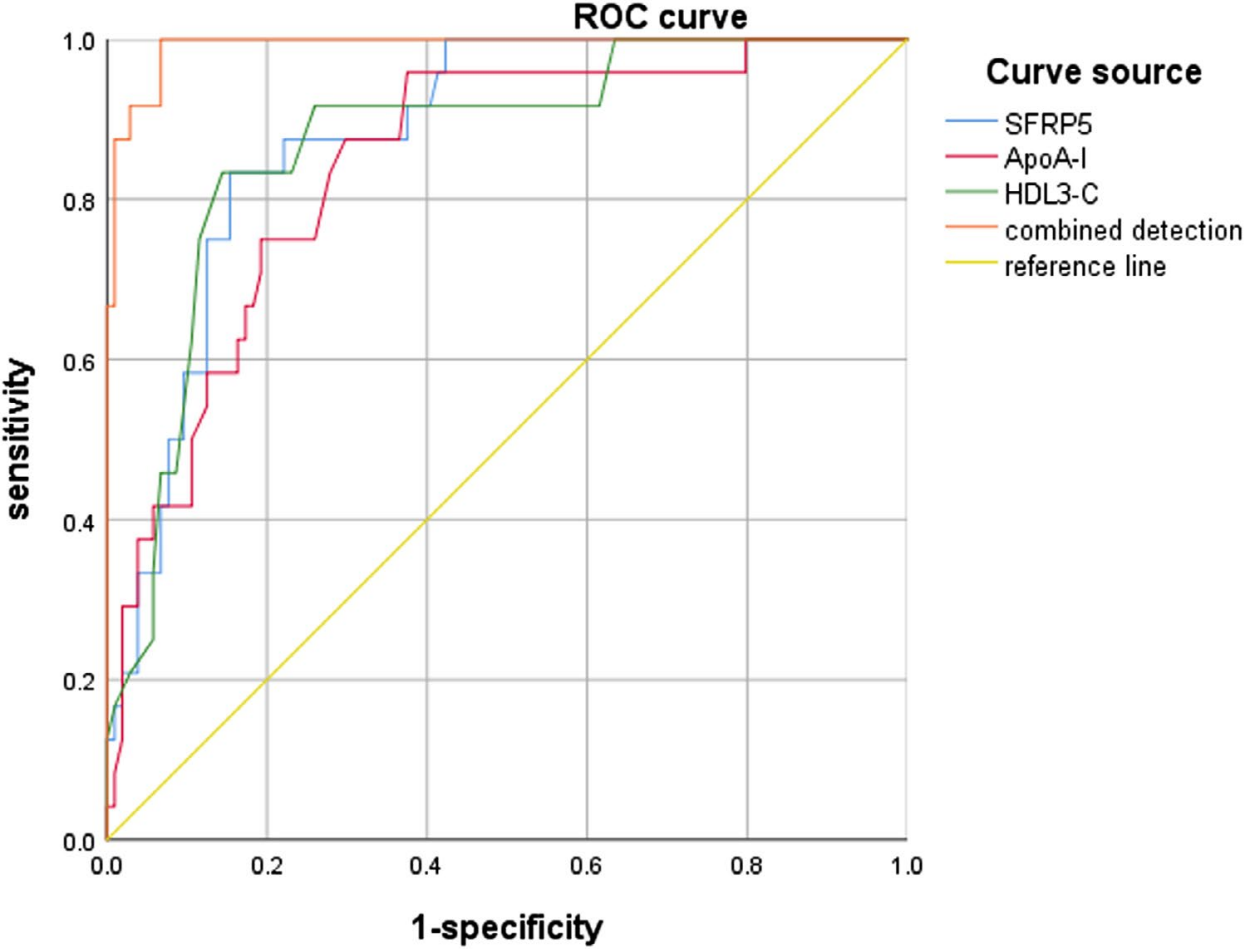
The predictive value of SFRP5, ApoA‐I, and HDL3‐C for ISR after PCI in AMI patients.

ROC curve showed that the AUC of SFRP5, ApoA‐I, and HDL3‐C levels alone and in combination to predict ISR in AMI patients after PCI was > 0.70, which had certain predictive value, and the combined value was higher. The AUC of combined detection was 0.991, indicating that the diagnostic value of combined detection was higher than that of the individual indicators (Table 6 and Figure 3).

**TABLE 6:** The predictive value of SFRP5, ApoA‐I, and HDL3‐C for ISR after PCI in AMI patients.

Indicators	AUC	Cut‐off value	95% CI	*p*	Specificity	Sensitivity	Yoden index
SFRP5	0.881	16.860 ng/mL	0.817–0.944	< 0.001	0.846	0.833	0.679
ApoA‐I	0.843	1.675 g/L	0.761–0.925	< 0.001	0.625	0.9580	0.583
HDL3‐C	0.871	0.345 mmol/L	0.795–0.948	< 0.001	0.856	0.833	0.689
Combined detection	0.991	—	0.980–1.000	< 0.001	0.933	1.000	0.933

## 4 Discussion

AMI is a common clinical cardiovascular emergency and severe disease, which is caused by the blockage of the heart blood supply channel for various reasons, and then myocardial necrosis caused by the imbalance of oxygen supply and demand [9]. The primary purpose of AMI treatment is to unblock the occluded coronary arteries and restore myocardial function. PCI is one of the most important treatment methods for patients with AMI. Timely and effective PCI can quickly unclog blood vessels, increase blood perfusion, and restore blood flow supply. However, PCI can damage the vascular endothelium, promote vascular smooth muscle cell hyperplasia and platelet aggregation, activate the coagulation system, and form thrombosis, resulting in the occurrence of ISR [10]. The results of this study showed that 24 out of 128 patients with AMI developed ISR after PCI, with an incidence rate of 18.75%. Although the incidence was not very high, ISR could cause symptoms such as chest pain, angina, difficulty breathing, palpitations, and so forth, which seriously affect the patient's life. Therefore, exploring the influencing factors of ISR occurrence in AMI patients after PCI is of great significance for reducing ISR occurrence and improving the prognosis of AMI patients.

The results of this study showed that the levels of SFRP5, ApoA‐I, and HDL3‐C in the ISR group were lower than those in the non‐ISR group. Meanwhile, binary logistic regression analysis showed that high expression of SFRP5, ApoA‐I, and HDL3‐C was a protective factor for ISR after PCI, and their levels were negatively correlated with the severity of ISR. Atherosclerosis is the key basis of ISR, and abnormal lipid metabolism is the main risk factor for atherosclerosis [11]. SFRP5 is involved in the pathogenesis of coronary heart disease and has anti‐inflammatory, endothelial cell protection, anti‐fibrosis, and other effects. It plays an important role in regulating the vascular inflammatory response and can also inhibit the formation and development of atherosclerotic plaques [12]. SFRP5 is mainly secreted by adipocytes and is an adipokine with cardiovascular protective effects [12]. Wnt5a can promote myocarditis and fibrosis, leading to the deterioration of myocardial remodeling after myocardial infarction. SFRP5 can inhibit the Wnt signaling pathway by binding to Wnt5a, activate nitric oxide synthase in endothelial cells, and relax blood vessels, thereby inhibiting the formation and development of atherosclerotic plaques, reducing the risk of ISR, and playing a protective role in cardiovascular diseases [13]. When the level of SFRP5 is increased, it can reduce the proliferation and differentiation of cardiac fibroblasts by regulating Wnt5a protein, accelerate the activation of cytokines, inhibit the activation of macrophages in adipose tissue, suppress the release of inflammatory cells, and reduce the degree of atherosclerosis to prevent ISR [14]. HDL‐C is a kind of lipoprotein with anti‐atherosclerosis properties. ApoA‐I, one of the main components of HDL‐C, is an anti‐atherosclerotic plaque formation factor and participates in the process of atherosclerosis [15]. ApoA‐I can catalyze lecithin, activate cholesterol acyltransferase, promote the transport of cholesterol from the vascular wall to the liver for metabolism and excretion, accelerate cholesterol metabolism, reduce excessive accumulation of cholesterol in the vascular wall, maintain normal blood lipid metabolism, and reduce the risk of atherosclerosis [16]. ApoA‐I plays an important role in the reverse cholesterol transport process and can also play an anti‐inflammatory and protective role in arterial vessels by increasing the expression of annexin A1 and inhibiting the activation of phospholipase A2 [17]. HDL3‐C is an important subtype of HDL‐C, which has the effects of reverse cholesterol transport, inhibition of low‐density lipoprotein cholesterol peroxidation, anti‐inflammation, and so on, and plays a better role in cardiovascular protection [18]. HDL3‐C can inhibit the formation of oxidized low‐density lipoprotein, prevent it from causing damage to endothelial cells and intracellular lipid accumulation, forming atherosclerotic plaques and inducing ISR [19]. These above results showed that SFRP5, ApoA‐I, and HDL3‐C played a protective role in cardiovascular disease, which could effectively reduce the degree of atherosclerosis, prevent the occurrence of ISR, and improve the degree of ISR. Therefore, the higher the levels of SFRP5, ApoA‐I, and HDL3‐C are, the lower the probability of ISR is and the degree of ISR is.

In addition to SFRP5, ApoA‐I, and HDL3‐C, diabetes and high levels of hs‐CRP are risk factors for ISR in AMI patients after PCI. ISR is closely related to vascular endothelial injury and intravascular inflammatory reactions. Stent implantation, vascular remodeling, and chronic inflammatory reactions of the vascular wall can destroy the functional integrity of the endothelial barrier, leading to the occurrence of ISR [20]. Inflammatory factors play an important role in the occurrence and development of atherosclerosis. Hs‐CRP is a common inflammatory marker that is deposited in the arterial wall with complement complexes and foam cells. Hs‐CRP is easy to bind to lipoproteins to activate the complement system, produce a large number of inflammatory factors, cause vascular endothelial damage, and aggravate lumen stenosis caused by atherosclerosis [21]. Diabetic patients have higher blood glucose, and their vascular endothelial function is impaired; local inflammation of endothelial cells is activated, platelets are activated after the exposure of vascular subcutaneous collagen tissue, and they accumulate at the damaged site. A variety of cytokines and growth factors are released, which promote the proliferation of smooth muscle cells and lead to local vascular stenosis [22]. High blood sugar can also stimulate collagen synthesis, leading to fibrosis of the vascular walls and loss of elasticity of the blood vessels, thereby increasing vascular resistance and pressure [23]. In previous studies, LDL‐C is an influencing factor for ISR after PCI. In this study, there was no difference in LDL‐C levels between the ISR group and the non‐ISR group. Due to the poor diet and living habits of the patients, which induced coronary heart disease, LDL‐C was at a poor level. Different stent types and drug regimens have different rates of ISR. Drug‐eluting stents can reduce the rate of restenosis and the incidence of late stent thrombosis compared with bare metal stents. Drug‐eluting stents were used in this study, and there was no difference in drug use and stent type between the two groups. According to Ouyang H et al. [24], there was no statistically significant difference in the incidence of ISR between patients using sirolimus and paclitaxel‐eluting stents, which was consistent with this study. The results of this study also showed that the postoperative medication of the patients was not related to the occurrence of ISR, which may be due to the fact that the postoperative medication of the patients referred to the relevant literature and the types of medication were relatively similar.

In this study, the ROC curve showed that the AUC of SFRP5, ApoA‐I, and HDL3‐C alone and in combination to predict the occurrence of ISR in AMI patients after PCI was greater than 0.70, which had certain predictive value, and the combined value was the highest. The results indicated that when the levels of SFRP5, ApoA‐I, and HDL3‐C were abnormally decreased, the risk of ISR was higher in AMI patients after PCI. Monitoring the levels of SFRP5, ApoA‐I, and HDL3‐C in AMI patients is helpful for early prediction of ISR. Patients with higher risk should be given cholesterol‐lowering drugs, such as niacin and fibrates, and instructed to have a low‐calorie diet, moderate exercise, weight control, and maintain a healthy lifestyle so as to prevent atherosclerosis and reduce the degree of ISR or prevent the occurrence of ISR.

In general, diabetes and high levels of hs‐CRP are risk factors for ISR in AMI patients after PCI. High levels of SFRP5, ApoA‐I, and HDL3‐C are protective factors for ISR after PCI and have certain predictive value. In clinical practice, we can pay close attention to the changes of the above indicators in AM patients and timely take corresponding measures to intervene to reduce the incidence of ISR in AMI patients after PCI. This study also had certain limitations. As a single‐center retrospective study, there is the possibility of selection bias, and the data may lack completeness. The sample size was relatively small (128 patients), which may affect the reliability and generalizability of the findings. Further multi‐center clinical validation is needed to ensure that the findings are applicable to a wider patient population. Moreover, this study only focused on SFRP5, ApoA‐I, HDL3‐C, diabetes, and hs‐CRP, and may have ignored other factors that may affect the development of ISR. In the future, internal and external validation of the risk prediction model should be carried out, and multi‐center and large sample clinical verification should be carried out.

## Conflicts of Interest

The authors declare no conflicts of interest.

## Data Availability

The data that support the findings of this study are available from the corresponding author upon reasonable request.
